# Impacts of Climate Changes on Spatiotemporal Variation of Cotton Water Requirement and Irrigation in Tarim Basin, Central Asia

**DOI:** 10.3390/plants13223234

**Published:** 2024-11-18

**Authors:** Min Xu, Hao Wu, Xiaoping Chen

**Affiliations:** 1 Key Laboratory of Cryospheric Science and Frozen Soil Engineering, Northwest Institute of Eco-Environment and Resources, Chinese Academy of Sciences, Lanzhou 730000, China; xumin@lzb.ac.cn; 2 University of Chinese Academy of Sciences, Beijing 100049, China; 3 College of Hydraulic Science and Engineering, Yangzhou University, Yangzhou 225001, China

**Keywords:** water requirement, irrigation, water profit and loss index, cotton, Tarim basin

## Abstract

Evapotranspiration (*ET_c_*), crop water requirement (*D_cr_*), irrigation (*IR*) and irrigation leaching amount (*IR_lc_*) play a critical role in optimizing irrigation scheduling and are also important for hydrological cycle processes and ecological environment in arid regions. This research examined the spatiotemporal variability of the *ET_c_*, *D_cr_* and *IR* of cotton using data from 16 meteorological stations in the Tarim basin (TRB) of arid Northwest China during 1961–2017. The results showed that the mean annual *ET_c_* of cotton exhibited a significant decreasing trend, with a change rate of 12.965 mm·10 a^−1^ and 18.357 mm·10 a^−1^ during 1961–2017 and 1961–1990, respectively. Subsequently, it experienced a substantial increase with a change rate of 16.833 mm·10 a^−1^ after 1990. The *D_cr_* of cotton followed a decreasing trend at a rate of 15.531 mm·10 a^−1^ and 21.99 mm·10 a^−1^ during 1961–2017 and 1961–1990, respectively. The *D_cr_* of cotton provided an increasing trend at a rate of 20.164 mm·10 a^−1^ during 1991–2017. The *IR* of cotton followed a decreasing trend at a rate of 19.66 mm·10 a^−1^ in 1961–2017 and 24.531 mm·10 a^−1^ in 1961–1990, but an increasing trend at a rate of 14.437 mm·10 a^−1^ in 1991–2017. The *IR_lc_* of cotton decreased by 2.566 mm·10 a^−1^ and 3.663 mm·10 a^−1^ during 1961–2017 and 1961–1990, respectively. After 1990, it experienced a substantial increase by 3.331 mm·10 a^−1^. Wind speed exerted the greatest influence on the variability in *D_cr_* and *IR* between 1961 and 1990, while shine hour played a more prominent role in explaining the variability in *D_cr_* and precipitation may have played a more significant role in explaining the variability in *IR*. This study is helpful for the scientific planning for agriculture, water resource allocation and water-saving irrigation in arid regions.

## 1. Introduction

The arid regions of Central Asia are highly vulnerable to water resources due to intensively irrigated agriculture, dry climate, precipitation deficit and strong evaporation [1,2]. Cotton (*Gossypium hirsutum* L.) is an economically important crop and has been widely cultivated in the Tarim basin (TRB), Central Asia [3]. The cotton planting area in this region accounts for over 70% of the total land, resulting in a substantial demand for irrigation water which contributes to approximately 6% of the agricultural water consumption. Consequently, the issue of water scarcity in agriculture is gaining increasing prominence [4]. In addition, the impact of climate change has resulted in alterations to regional hydrological processes and the spatiotemporal distribution of available water resources. Consequently, this has exacerbated the agricultural water crisis. Jans et al. [5] reported that the demand of irrigation water for cotton may be challenged by climate change in future. Noteworthy, saline land is one of the main land types in the planted cotton area in the Tarim basin. These factors have further exacerbated the shortage of water for cotton in the region. Therefore, it is necessary to focus on quantifying irrigation water requirements for cotton under climate change in the Tarim basin.

Crop evapotranspiration (*ET_C_*) is the key parameter for calculating crop water requirements, which combine water loss due to evaporation from the soil surface and transpiration from the crop. This can be observed by various methods, including eddy covariance systems, large aperture scintillometer, water balance methods, agricultural systems models, the crop coefficient method and lysimeters. Using a large-scale lysimeter, Liu and Qiao [6] showed that the average evapotranspiration of cotton fields during the whole growth period under no-mulching and mulching was 429.22 mm and 292.15 mm in Xinjiang, respectively. Numerous studies [7,8] reported on the effect of different management measures on *ET_c_* at different growth stages in the TBR and its relationship with climate variables using a large-scale lysimeter. The models of agricultural systems are effective tools for evaluating the impact of climate variables on water requirement and yield of cotton. Chen et al. [9] reported that the average cotton water requirement based on simulations during sowing to maturity in 1970–2000 was 786 mm in the TRB. Also, Yang et al. [10] simulated the water requirement of cotton and showed that it ranged from 598 mm to 633 mm in 1961–1990. However, those methods are limited to a certain region and cannot be analyzed on a spatiotemporal scale. Based on the crop coefficient method, Su et al. [11] analyzed the spatiotemporal changes in cotton *ET_c_* in China from 1960 to 2019 and reported that the rate of decline for cotton *ET_c_* decreased from 916.25 mm to 886.74 mm in inland cotton regions of the Northwest China. However, the amount of irrigation water used to wash salt before sowing should not be neglected in the TRB when calculating cotton water requirements.

Soil salinization is another major factor limiting cotton production in the TRB. Benouniche et al. [12] reported that additional leaching water was needed to remove soil salinity in the root zone. Although cotton has a certain salinity tolerance, the key measure to ensure cotton yield was to reduce soil salinity levels by leaching irrigation during the non-growth period [13,14]. Yu et al. [15] indicated that the relative yield of cotton could be maintained at 85–50% when the soil salt content is 0.450–0.581%. Flood irrigation is the most common leaching method, so leaching irrigation amount and timing are extremely important in salinization and water shortage regions. Zhang et al. [16] reported that an irrigation amount of 3600 m^3^ hm^−2^ in winter was beneficial to cotton sowing and emergence after spring in Xinjiang. However, Hu et al. [17] have shown that salt leaching in the growth period instead of flood irrigation in the non-growth period can improve the salt leaching effect and reduce the risk of secondary salt. Also, Liu et al. [18] reported that increasing the leaching irrigation amount first increased and then decreased the seed cotton yield, and reported on the optimal seed cotton yield under a leaching amount of 150 mm at the seedling and budding stages in saline–alkaline soils. In recent years, compared with field control experiments, crop models have been widely used to optimize the leaching irrigation amount due to the advantages of high efficiency and easy control of climate variables. Using the Aquacrop model, Song et al. [19] recommended a total leaching irrigation amount of 80%*ET_c_* + 120 mm in wet years and 100%*ET_c_* + 120 mm in normal and dry years in cotton fields in the TRB. However, it is difficult for the Aquacrop model to evaluate the change in irrigation leaching irrigation amount on a spatial scale.

The Tarim basin in Central Asia is an extremely arid area and is an important cotton production base in China. The raised groundwater level of the Tarim basin and secondary salinization of the soil occurred due to improper irrigation and low anti-seepage measures. In addition, changes in recent decades from a warm/dry to a warm/wet climate have led to modifications in regional hydrological processes and the spatiotemporal distribution of available water resources for agricultural production. In previous studies, the water requirement of cotton at each growth stage and the whole growth period was evaluated by the crop coefficient method in this region [20]. However, the impact of climate change on irrigation water requirements of cotton was less considered in saline–alkali soils. The present study was designed to predict the impact of climate change on the spatiotemporal variation of cotton water requirements and leaching irrigation in the Tarim basin. Therefore, the specific objectives of study were as follows: (*i*) to evaluate the evapotranspiration, water requirement, irrigation and leaching irrigation water of cotton under warm/dry to warm/wet climate patterns, and (*ii*) to quantify the effects of climate change on water requirements and the irrigation of cotton under warm/dry to warm/wet climate patterns.

## 2. Results

### 2.1. Spatiotemporal Characteristics of Temperature and Precipitation

**Figure 1:**
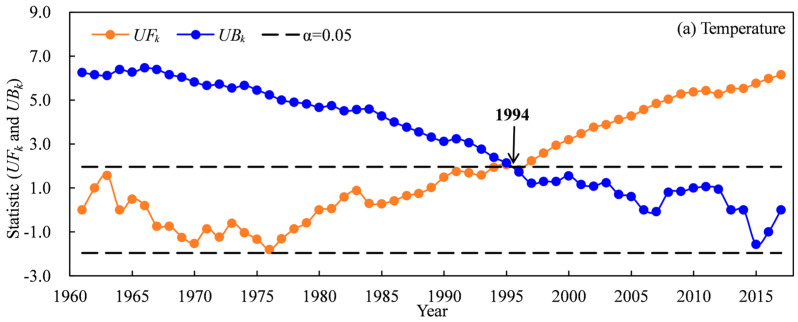

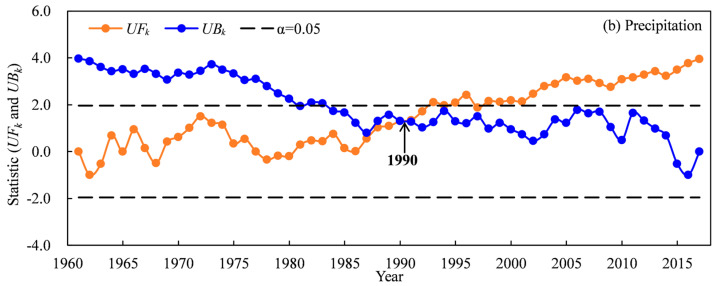
Mann–Kendall abrupt change test of temperature (**a**) and precipitation (**b**) in the Tarim basin during 1961–2017.

**Figure 2:**
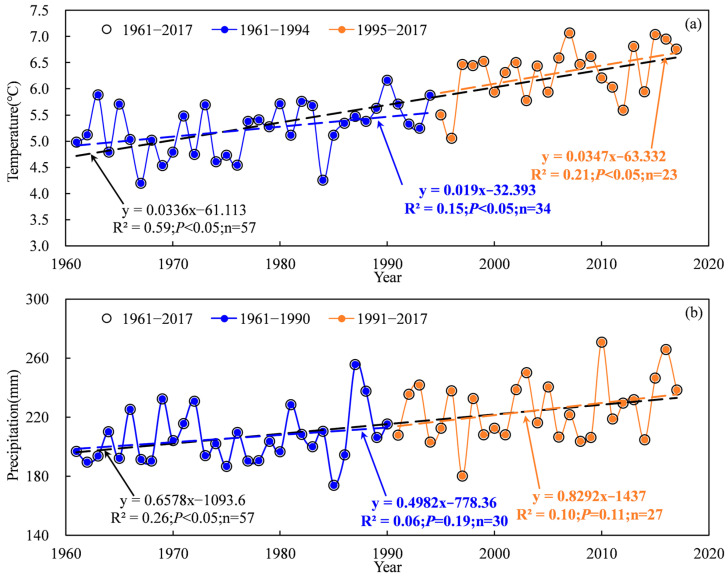
Variations in mean annual temperature (**a**) and precipitation (**b**) in the Tarim basin during 1961–2017.

**Figure 3:**
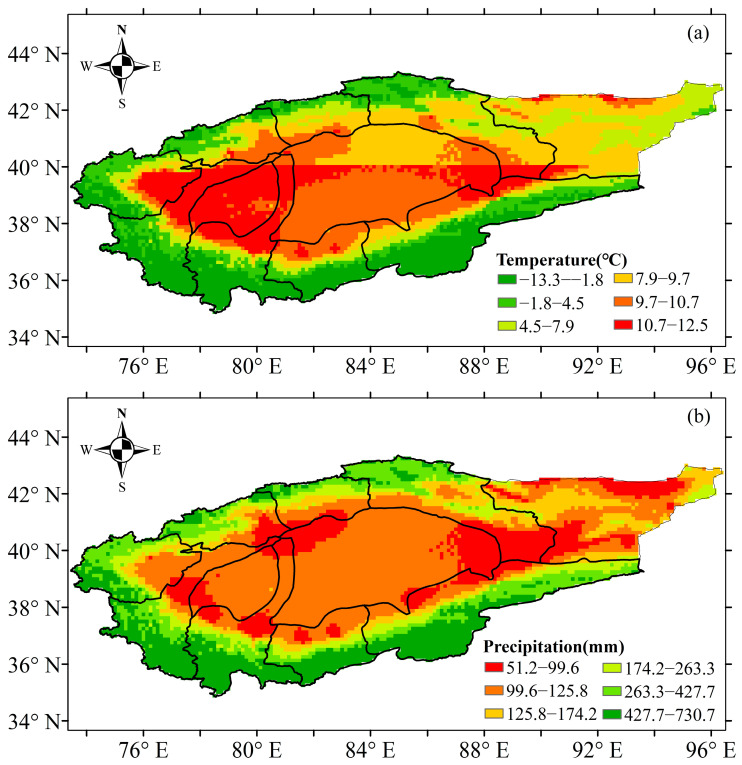
Spatial patterns of mean annual temperature (**a**) and precipitation (**b**) in the Tarim basin during 1961–2017.

The temporal scale changes of temperature and precipitation based on national meteorological stations during 1961–2017 are shown in Figure 1 and Figure 2. The Mann–Kendall abrupt change test suggested that the abrupt change point of annual temperature occurred in 1994 for the TRB (Figure 1a). The mean annual temperature showed a significant increasing trend in the TRB, which increased obviously after 1994 (Figure 2a). The mean annual temperature in the TRB ranged from 4.19 °C to 7.07 °C during 1961–2017. The temperature in 1961–2017, 1961–1994 and 1995–2017 increased by 0.336 °C·10 a^−1^, 0.190 °C·10 a^−1^ and 0.347 °C·10 a^−1^ in the TRB, respectively (Figure 2a). The Mann–Kendall abrupt change test also suggested that the abrupt change point of annual precipitation occurred in 1990 for the TRB (Figure 1b). A significant increasing trend in precipitation was also found in the TRB, with an increase of 6.578 mm·10 a^−1^ during 1961–2017 (Figure 2b). Precipitation in the 1961–1990 and 1991–2017 periods showed no significant increase, and increased by 4.982 mm·10 a^−1^ and 8.292 mm·10 a^−1^, respectively (Figure 2b). The spatial distribution of the average temperature in the TRB showed that the lowest (−13.3–−1.8 °C) and highest (10.7–12.5 °C) temperatures occurred upstream and downstream the river (Figure 3a). In the cotton-planted areas, the average annual temperature was mostly above 4.5 °C. In contrast, the downstream areas of the Aksu, Hoten and Keriya rivers had the lowest (51–100 mm) precipitation in the TRB (Figure 3b).

### 2.2. Changes in the Evapotranspiration and Water Requirement of Cotton

**Figure 4:**
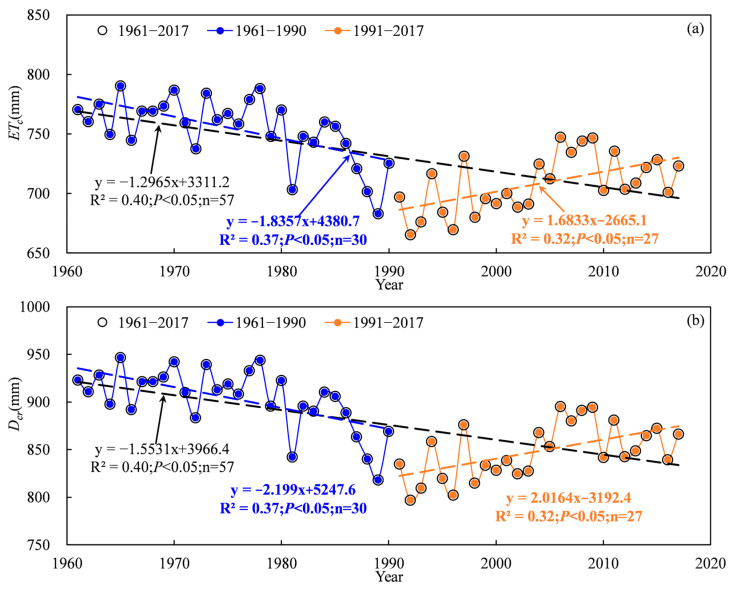
Variations in evapotranspiration (**a**) and water requirements (**b**) of cotton in the Tarim basin during 1961–2017.

**Figure 5:**
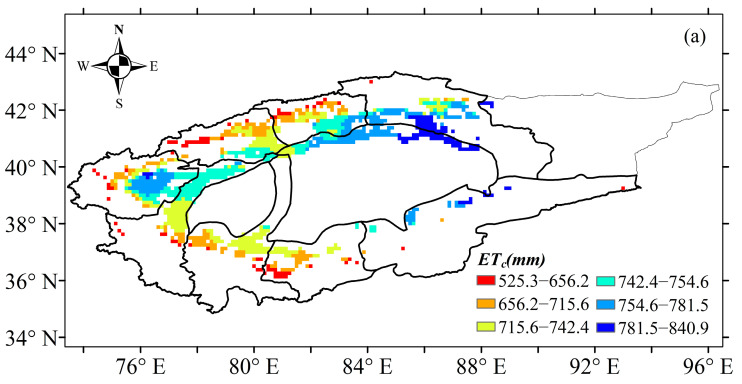

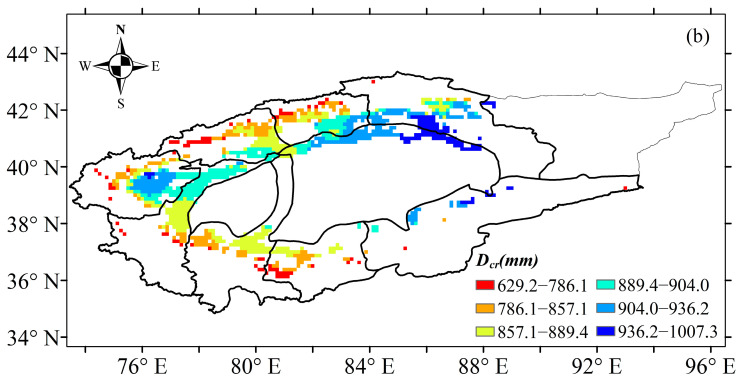
Spatial patterns of evapotranspiration (**a**) and water requirements (**b**) of cotton in the Tarim basin during 1961–2017.

Based on the abrupt change point of annual precipitation, the evapotranspiration and time series of cotton water requirements were divided into three periods: 1961–2017, 1961–1990 and 1991–2017. The temporal scale characteristics of the evapotranspiration and cotton water requirements during 1961–2017 are shown in Figure 4 and Figure 5. A significantly decreasing trend for evapotranspiration and cotton water requirement in the TRB were found at the interannual scale, with a change rate of 12.965 mm·10 a^−1^ and 15.531 mm·10 a^−1^, respectively (Figure 4 and Figure 5). The mean annual evapotranspiration and water requirement of cotton in the TRB ranged from 665.4 mm to 790.4 mm and 797.0 mm to 946.9 mm during 1961–2017, respectively. Results indicated that the cotton annual evapotranspiration in the TRB showed a significant decreasing trend with a change rate of 18.357 mm·10 a^−1^ from 1961 to 1990, which increased significantly with a rate of 16.833 mm·10 a^−1^ after 1990 (Figure 4a). The annual cotton water requirement in the TRB also showed a significant decreasing trend with a rate of 21.99 mm·10 a^−1^ from 1961 to 1990, which increased significantly with a rate of 20.164 mm·10 a^−1^ after 1990 (Figure 4b). The spatial distribution of the average cotton evapotranspiration showed obvious and varying differences in the TRB, increasing from the west to the east and the south to the north of the basin (Figure 5a). The Kaidu river basin provided the highest cotton evapotranspiration, ranging from 781.5 mm to 840.9 mm. The lowest cotton evapotranspiration ranged from 525.3 mm to 656.2 mm in the Aksu and Keriya river basins. The spatial distribution characteristics of cotton water requirements were similar to cotton evapotranspiration, ranging from 629.2 mm to 1007.3 mm (Figure 5b).

### 2.3. Changes in the Irrigation of Cotton

**Figure 6:**
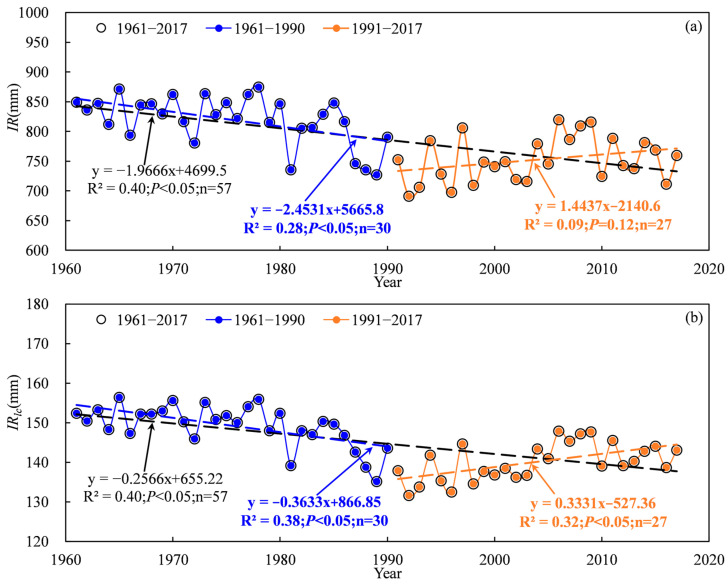
Variations in irrigation water use (**a**) and the irrigation leaching amount (**b**) of cotton in the Tarim basin during 1961–2017.

**Figure 7:**
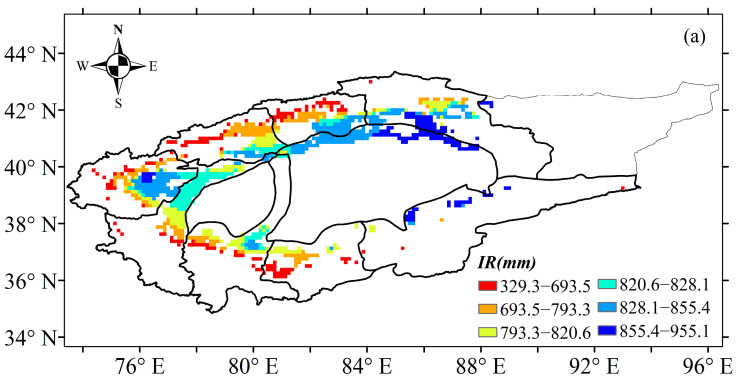

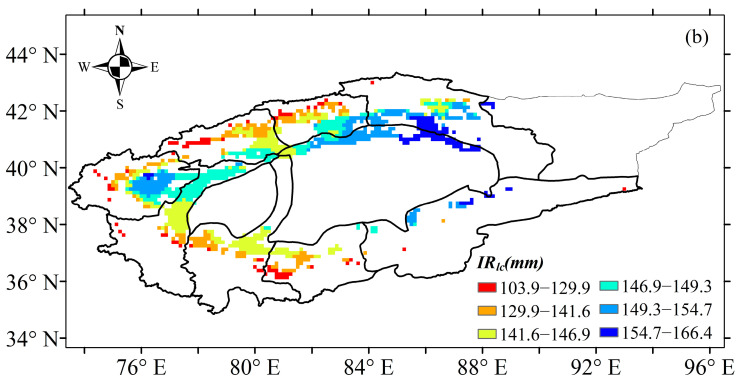
Spatial patterns of irrigation water use (**a**) and the irrigation leaching amount (**b**) in the Tarim basin during 1961–2017.

Based on the abrupt change point of annual precipitation, the irrigation of cotton time series was divided into three periods: 1961–2017, 1961–1990 and 1991–2017. Figure 6 shows the temporal trends of irrigation water use and the irrigation leaching amount in the TRB from 1961 to 2017. Results showed that irrigation water use and the irrigation leaching amount of cotton followed a significant decreasing trend during 1961–2017 (Figure 6). The water use and leaching amount of irrigation in the TRB ranged from 690.1 mm to 874.9 mm and 131.7 mm to 156.4 mm, respectively. The irrigation water use of cotton in the TRB showed a significant decreasing trend from 1961 to 1990 but no significant increasing trend from 1991 to 2017 (Figure 6a). In contrast, the irrigation leaching amount followed a significant decreasing trend during 1961–1990 but a significant increasing trend from 1991 to 2017 (Figure 6b). Water use and the leaching amount of cotton decreased by 19.66 mm·10 a^−1^ and 2.566 mm·10 a^−1^ during 1961–2017, respectively (Figure 6). In the 1961–1990 period, a decrease of 24.531 mm·10 a^−1^ and 3.663 mm·10 a^−1^ was found water use and the leaching amount of irrigation, respectively (Figure 6). After 1990, water use and the leaching amount of cotton increased by 14.437 mm·10 a^−1^ and 3.331 mm·10 a^−1^, respectively (Figure 6). On a spatial scale, the irrigation water use of cotton increased from the west to the east and the south to the north of the TRB, ranging from 329.3 mm to 955.1 mm (Figure 7a). The highest water use (855.4–955.1 mm) and leaching amount (154.7–166.4 mm) of irrigation occurred in the Kaidu river basin. Also, the Aksu and Keriya river basin had the lowest water use and leaching amount of irrigation. A similar spatial distribution of the leaching amount of irrigation to irrigation water use in the TRB occurred (Figure 7b).

### 2.4. Changes in Water Profit and Loss Index

**Figure 8:**
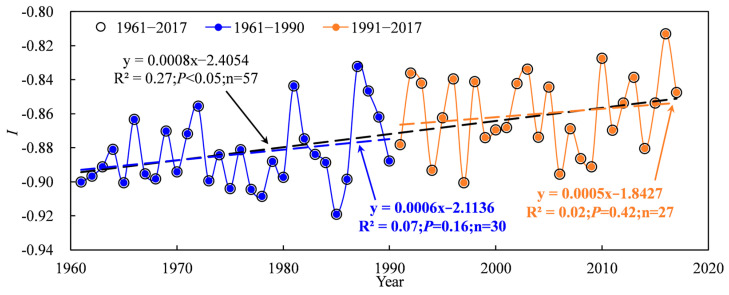
Variations in water profit and loss index of cotton in the Tarim basin during 1961–2017.

**Figure 9:**
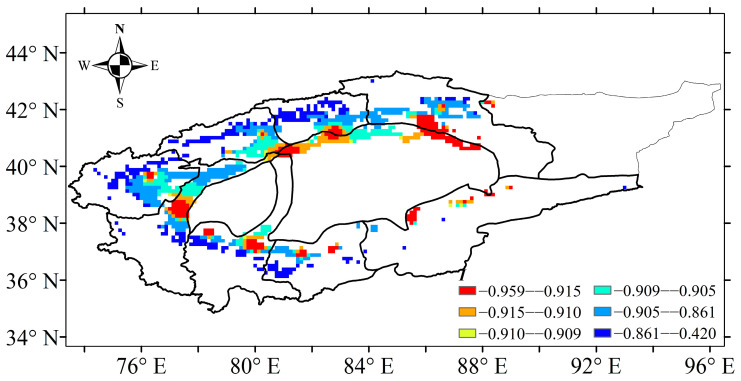
Spatial patterns of water profit and loss index of cotton in the Tarim basin during 1961–2017.

Based on the abrupt change point of annual precipitation, the water profit and loss index of cotton time series was also divided into three periods: 1961–2017, 1961–1990 and 1991–2017. Figure 8 shows the water profit and loss index in the TRB in 1961–2017. Results showed that cotton in the TRB was in the state of water deficit at each growth stage, with a negative value (−0.92 < *I* < −0.81) of water profit and loss index over 1961–2017. However, a significant increasing trend in the water profit and loss index during 1961–2017 was found, with an increase of 0.008 10 a^−1^ (Figure 8). There was no significant increase in the water profit and loss index during the 1961–1990 and 1991–2017 periods (Figure 8). On a spatial scale, the average water profit and loss index decreased from the west to the east and the south to the north of the TRB, ranging from −0.42 to −0.959 (Figure 9). The lowest water profit and loss index (−0.959 to −0.915) occurred in the downstream areas of the Kaidu river basin, Keriya river basin, Hotan river basin and Aksu river basin.

## 3. Discussion

### 3.1. The Effects of Climate Change on Water Requirement and Irrigation of Cotton

**Figure 10:**
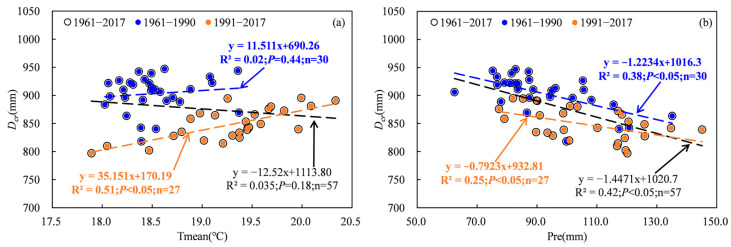

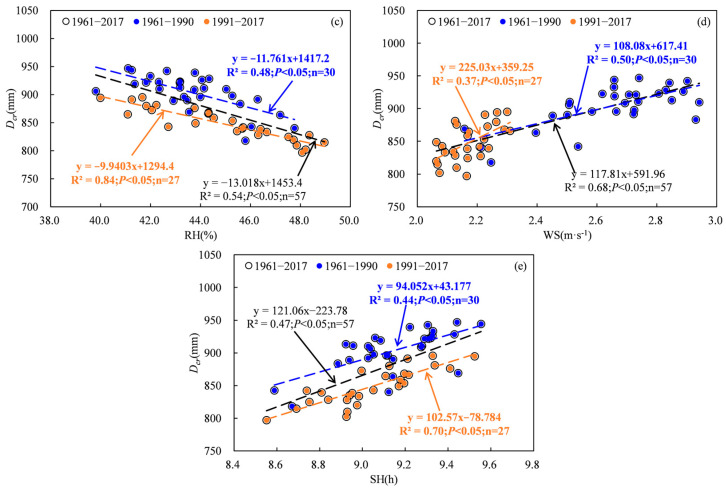
Relationships between water requirement and meteorological factors. (**a**), Tmean; (**b**), *Pre*; (**c**), *RH*; (**d**), WS; (**e**), SH.

**Figure 11:**
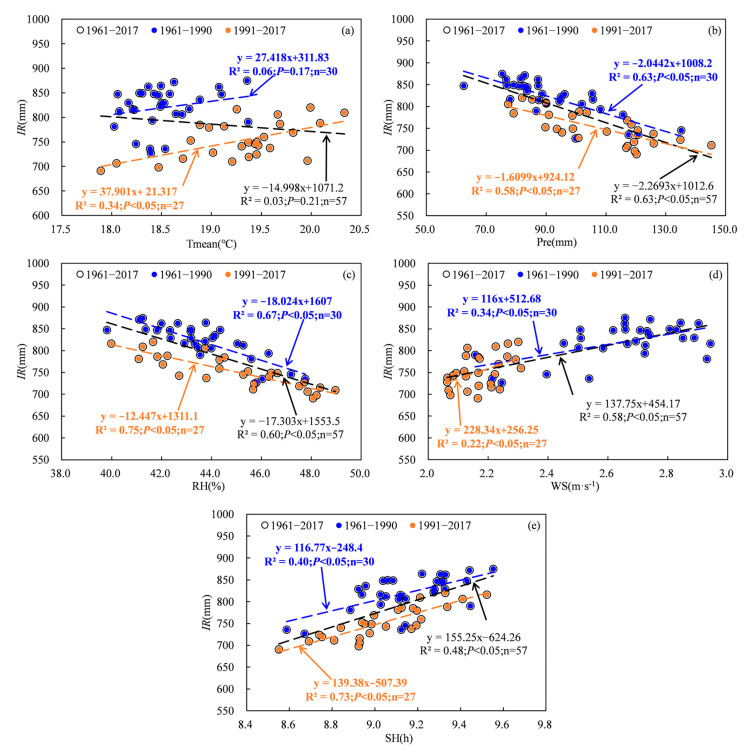
Relationships between irrigation and meteorological factors. (**a**), Tmean; (**b**), *Pre*; (**c**), *RH*; (**d**), WS; (**e**), SH.

The water requirement and irrigation of cotton are greatly impacted by meteorological factors, particularly in arid regions [9,21,22]. Previous studies have indicated a positive correlation between temperature, wind speed, sunshine hours and water requirement and irrigation, as well as a negative correlation between relative humidity and precipitation and water demand and irrigation [3,22,23]. The results in this study were consistent with previous studies. Statistical analysis revealed significant negative correlations between *IR*, *D_cr_* and *RH* and *Pre*, as well as significant positive correlations between *IR*, *D_cr_* and WS and SH (Figure 10 and Figure 11). The relationships between *IR* and Tmean showed a significant positive correlation (Figure 10 and Figure 11). Although the correlation between *D_cr_* and Tmean was not statistically significant for the period 1961 to 2017, it exhibited a non-significant negative correlation for 1961 to 1990 and a significant positive correlation for 1991 to 2017 (Figure 10). Partial correlation analysis revealed that the absolute values of the partial correlation coefficients between WS and *D_cr_* and *IR* were highest during 1961–1990, while those between SH and *D_cr_* as well as *Pre* and *IR* were highest during 1991–2017 (Table 1). This means that WS may have exerted the greatest influence on the variability in *D_cr_* and *IR* between 1961 and 1990, while SH may have played a more prominent role in explaining the variability in *D_cr_*, and *Pre* may have played a more significant role in explaining the variability in *IR* from 1991 to 2017.

### 3.2. The Effects of Drought on Water Requirement and Irrigation of Cotton

**Figure 12:**
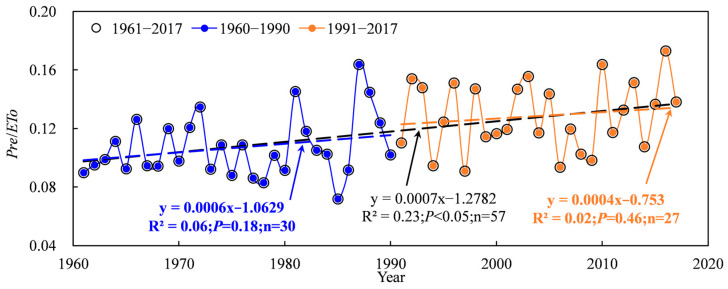
Variations in *Pre*/*ETo* during 1961–2017 in the Tarim basin.

**Figure 13:**
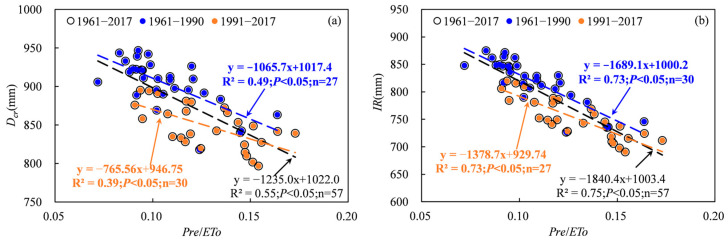
Relationships between water requirement (**a**), irrigation (**b**) and *Pre*/*ETo*.

The Tarim basin is a desert region characterized by an arid climate and precipitation deficiency, and the extent of drought significantly influences water requirements and cotton irrigation [24,25]. The severity of drought can be quantified by the ratio of precipitation to evapotranspiration (*Pre*/*ETo*), and this ratio exhibited a significant increasing trend from 1961 to 2017 (Figure 12). The results indicate that climate change induced a decreased drought degree. In addition, the increased rate of *Pre*/*ETo* in 1991–2017 was lower than that in 1961–1990, which suggests that the climate change from warm/dry to warm/wet in the 1990s led the extent of decrease in drought reduction. However, the state of drought remained unchanged, with *Pre*/*ETo* values ranging from 0.07 to 0.17 (Figure 12). The relationships between *IR*, *D_cr_* and *Pre*/*ETo* exhibited a significant negative correlation (Figure 13). The above results showed that irrigation and the cotton water requirement decreased with a decreasing degree of drought. The mean annual precipitation in the Tarim basin showed a significant increasing trend during 1961–2017, which increased more obviously after 1990 (Figure 1b and Figure 2b). *Pre*/*ETo* showed a significant increasing trend from 1961–2017. These results further indicated that the decreased irrigation and cotton water requirements were induced by increasing precipitation.

## 4. Methods and Data

### 4.1. Study Area

**Figure 14:**
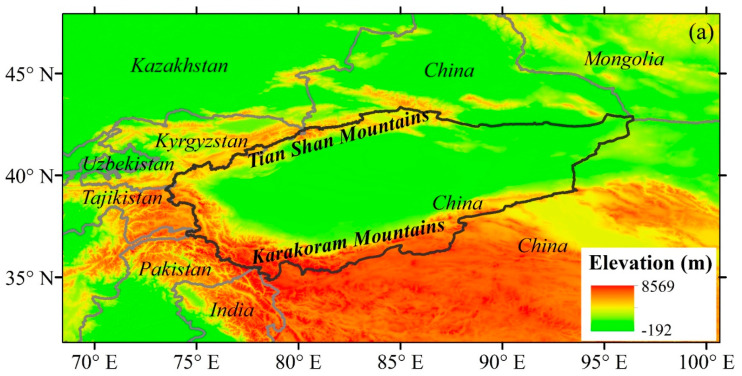

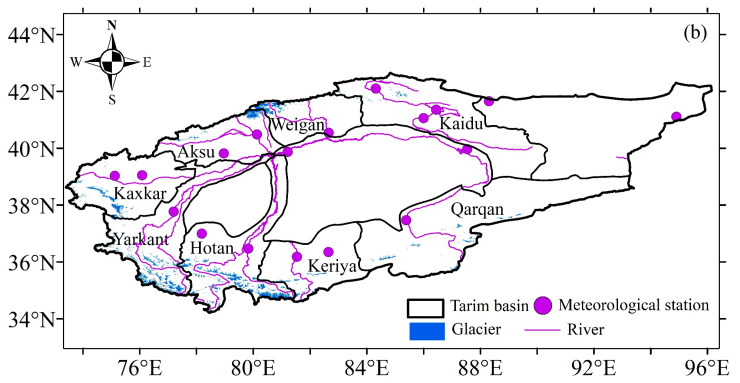
Location of the Tarim River basin (**a**) and distributions of glaciers, meteorological stations and rivers in the study area (**b**).

The Tarim basin (TRB), encompassing a total area of approximately 1.02 × 10^6^ km^2^, is situated in the southern region of Xinjiang, western China (34°–45° N; 73°–97° E) (Figure 14). Precipitation is greater than 300 mm in the mountainous regions and below 50 mm in the lower basin, and more than 54% of precipitation occurs in June, July and August. The temperature in the basin exhibits significant seasonal variations with an average air temperature of 7.6 °C, ranging from −35 °C in winter to 40 °C in summer [26,27]. The evaporation is highly intense, averaging 2500–3400 mm. The basin is encompassed by the eastern Pamir, Kunlun, Tianshan and Karakorum mountains, with elevations ranging from −156 to 8238 m. It comprises eight river basins, including Kaxkar, Aksu, Weigan, Yarkant, Keriya, Hotan, Qarqan and Kaidu. Water supply from mountains to the TRB prevails. The glacier/snow meltwater accounts for 38.5%, where discharge is mainly generated by glacier/snow meltwater and precipitation in upper mountainous regions [28]. The study area is an important irrigated agricultural area with low irrigation availability and high soil salinity. As a drought-tolerant crop, cotton is suitable for growing in this region, where planting area has gradually increased.

### 4.2. Dataset

Climate data were collected from China Meteorological Data Service Centre (http://data.cma.cn/; accessed on 6 December 2023). Sixteen meteorological stations monitored relative humidity (*RH*), minimum and maximum temperature (T_max_, T_min_), wind speed (WS), precipitation (*Pre*), sunshine hours (SH), latitude, longitude and elevation (Figure 14). The study period was from 1961 to 2017. Time-series plotting and inspection were applied to assess possible errors and uncertainties in dataset [29,30]. The corresponding long-term mean values were used to fill in missing data [29]. The WS, *RH* and SH were interpolated to each grid cell using the inverse distance weighting method. The minimum and maximum temperature and precipitation were interpolated to each grid cell by using gradient-plus-inverse distance weighting method [31].

### 4.3. Methods

#### 4.3.1. Calculation of Cotton Water Requirement

The study area predominantly consists of saline–alkali soil, necessitating that the crop water requirement encompasses both crop evapotranspiration consumption and leaching water demand [32]. The main formula is defined as follows:

(1) $$D_{cr}=\frac{ET_{c}}{1-L_{cr}}$$

(2) $$L_{cr}=\frac{EC_{i}}{5EC_{t}-EC_{i}}$$

where *D_cr_* is cotton water requirement (mm), *EC_i_* is electric conductivity of irrigation (dS·m^−1^) and *EC_t_* is salinity tolerance threshold of cotton (dS·m^−1^). *ET_c_* is evapotranspiration of cotton (mm), which was calculated by crop coefficient method; the main formula is defined as follows:

(3) $$ET_{c}=K_{c}\times ET_{o}$$

(4) $$ET_{0}=\frac{0.408\left(R_{n}-G\right)+\gamma \frac{900}{T+273}u_{2}\left(e_{a}-e_{d}\right)}{\Delta +\gamma \left(1+0.34u_{2}\right)}$$

where *ET_c_* is evapotranspiration of cotton (mm), Δ is slope vapor pressure curve (kPa·°C^−1^), *G* is soil heat flux density (MJ·m^−2^·d^−1^), *T* is mean daily air temperature at 2 m height (°C), *R_n_* is net radiation at the crop surface (MJ·m^−2^·d^−1^), *u*_2_ is wind speed at 2 m height (m·s^−1^), *e_a_* is saturation vapor pressure (kPa), *e_d_* is actual vapor pressure (kPa), *ET_o_* is the reference crop evapotranspiration (mm), *γ* is psychrometric constant (kPa·°C^−1^) and *K_c_* is crop coefficient determined. The specific values of *K_c_* at different growth stages were calculated as follows [3]:

(5) $$K_{cini}=K_{cini\left(Tab\right)}+\left[0.04\left(u_{2}-2\right)-0.004\left(RH_{\min}-45\right)\right]{\left(\frac{h}{3}\right)}^{0.3}$$

(6) $$K_{cmid}=K_{cmid\left(Tab\right)}+\left[0.04\left(u_{2}-2\right)-0.004\left(RH_{\min}-45\right)\right]{\left(\frac{h}{3}\right)}^{0.3}$$

(7) $$K_{cend}=K_{cend\left(Tab\right)}+\left[0.04\left(u_{2}-2\right)-0.004\left(RH_{\min}-45\right)\right]{\left(\frac{h}{3}\right)}^{0.3}$$

(8) $$K_{cdev}=K_{cprev}+\frac{i-\sum L_{cprev}}{L_{cstage}}\left(K_{cnext}-K_{cprev}\right)$$

where *K_cini_*, *K_cdevi_*, *K*_cmid_ and *K_cend_* are the coefficients of crop at the initial, crop development, mid-season and end of the late-season stages, respectively. *K_c__ini_*_(*Tab*)_, *K_cmid_*_(*Tab*)_ and *K_cend_*_(*Tab*)_ are the typical values for *K_cini_*, *K_cmid_* or *K_cend_* for cotton recommended by FAO-56. *K_cnext_* and *K_cprev_* are the *K_c_* values at the beginning of the next stage and the end of the previous stage, respectively. *u*_2_, *RH*_min_ and *h* are the mean values for daily wind speed at a height of 2 m, minimum relative humidity (%) and crop height (m), respectively. *i* is the day number within the growing season. *L_cprev_* is the length of all previous stages (days). *L_cstage_* is the length of the stage under consideration (days).

#### 4.3.2. Calculation of the Cotton Irrigation and Water Profit and Loss Index

Irrigation is calculated from crop water requirements and effective rainfall [33]. The main formula is defined as follows:

(9) $$IR=D_{cr}-P_{e}$$

(10) $$IR_{lc}=D_{cr}\cdot L_{\mathrm{cr}}$$

(11) $$P_{e}=\left\{\begin{array}{ll} P\left(4.17-0.2P\right)/4.17 & \left(P\leq 8.3\mathrm{mm}\right)\\ 4.17+0.1P & \left(P>8.3\mathrm{mm}\right) \end{array}\right.$$

where *IR* is irrigation (mm), *IR_lc_* is leaching irrigation (mm), *P_e_* is daily precipitation (mm) and *P_e_* is effective precipitation (mm).

Based on the effective precipitation and evapotranspiration, the water profit and loss index was calculated. The main formula is expressed as follows:

(12) $$I=\frac{P_{e}-ET_{c}}{ET_{c}}$$

where *I* is water profit and loss index (mm). The value of *I* exceeding 0 indicates an excess of water, while a value of *I* less than 0 indicates a deficit in water.

#### 4.3.3. Mann–Kendall Abrupt Change Test

The nonparametric Mann–Kendall abrupt change test was widely applied to detect the mutation points of hydrometeorological time series [34,35,36]. The null hypothesis (H_0_) assumes that there is no significant increasing or decreasing trend in the time series. The time series has a significant variation trend based on the alternative hypothesis. The main formula is defined as follows [34]:

The rank sequence *S_k_* of time series *X* with sample size *n*:

(13) $$S_{k}=\sum_{i=1}^{k}r_{i},\left(k=2,3,\ldots ,n;\;i=1,2,\ldots ,k\right)$$

(14) $$r_{i}=\left\{\begin{array}{l} 1,\begin{array}{cc} & X_{i}>X_{j} \end{array}\\ 0,\begin{array}{cc} & X_{i}<X_{j} \end{array} \end{array}\right.,\;j=1,2,\ldots ,i$$

Then, the statistics corresponding to sequential time series are calculated:

(15) $$UF_{k}=\frac{S_{k}-E\left(S_{k}\right)}{\sqrt{\mathrm{var}\left(S_{k}\right)}},k=2,3,\ldots ,n$$

where *UF*_1_ = 0 and *E*(*S_k_*) and var(*S_k_*) are the mean and variance of *S_k_*, respectively. Main formula is expressed as follows:

(16) $$E\left(S_{k}\right)=\frac{k\left(k-1\right)}{4},k=2,3,\ldots ,n$$

(17) $$\mathrm{var}\left(S_{k}\right)=\frac{k\left(k-1\right)\left(2k+5\right)}{72},k=2,3,\ldots ,n$$

The corresponding *S_k_* of the time series in the case of the inverse sequence was calculated. To obtain the inverse time series *X*′, the time series *X* is inverted, while letting

(18) $$UB_{k}=-UF_{k},k=n,n-1,\ldots ,1,UB_{1}=0$$

The standardized statistic *UF_k_* can be approximated by a normal distribution under the assumption of large sample conditions (*n* > 10). Therefore, when α = 0.05, *U*_0.05_ equals 1.96. Given the significance level α (α = 0.05 in this study), the curves of *UF_k_* and *UB_k_* and the straight lines of the critical values of *α* are drawn on a graph. If |*UF_k_*| > *U_α_*, it indicates a significant change trend in the time series during the observation period. When the curves of *UF_k_* and *UB_k_* intersect between the two critical value lines, the time corresponding to the intersection point is the start time of the abrupt change.

#### 4.3.4. Climate Tendency Rate

The correlation between meteorological factors and time can be quantified through a linear regression equation. The main formula is defined as follows:

(19) y=at+b

where *y* is meteorological elements. *t* is the time corresponding to *y*. *a* and *b* are regression coefficients. When *a* > 0, it indicates an increasing trend in *y*. Conversely, when *a* < 0, it suggests a decreasing trend in *y*. The significance level of 0.05 is used in the analysis of variance to determine whether there is a significant change trend. If the *p*-value is less than 0.05, it indicates a statistically significant change trend; otherwise, the change trend is not considered statistically significant. Additionally, *a* × 10 represents the climate tendency rate per decade for *y*.

#### 4.3.5. Partial Correlation Analysis

To assess the relationship between two elements and excludes the influences of other elements, partial correlation analysis was used [35,37]. Partial correlation analysis is used to investigate inherent relations while excluding the effects of controlling random variables [38]. The main formula is defined as follows:

(20) $$R_{xy\cdot z}=\frac{R_{xy}-R_{xz}\cdot R_{yz}}{\sqrt{\left(1-R_{xz}^{2}\right)\left(1-R_{yz}^{2}\right)}}$$

where *R_xy__·z_* is the partial correlation coefficient between *x* and *y*, with *z* as a controlling variable. *R_xy_*, *R_xz_* and *R_yz_* are the correlation coefficients between *x*, *y* and *z*, respectively. In the two-tailed *T* test, a significance level of *p* < 0.05 indicates a statistically significant correlation, while a significance level of *p* < 0.01 suggests an even stronger statistical significance; otherwise, the correlation is deemed not statistically significant.

#### 4.3.6. Software Tools

In this study, the cotton water requirement, evapotranspiration, irrigation, leaching irrigation, water profit and loss index, Mann–Kendall abrupt change test, climate tendency rate and partial correlation analysis were calculated by using self-programming in the MATLAB software (R2021a). The graphics were generated using the software tools of Excel 2019 and ARCGIS 10.7.

## 5. Conclusions

The evapotranspiration (*ET_c_*), water requirement (*D_cr_*), irrigation (*IR*) and water profit and loss index (*I*) of cotton were estimated in the Tarim basin (TRB). The spatiotemporal distribution of the water requirement (*D_cr_*), irrigation (*IR*), irrigation leaching amount *(IR_lc_*) and water profit and loss index (*I*) were investigated. In addition, a partial correlation analysis was applied to identify the effects of climate change on water requirement and cotton irrigation. The main conclusions were drawn as follows.

The mean annual precipitation showed a significant increasing trend during 1961–2017 and obviously increased after 1991 in the TRB. The mean annual temperature also significantly increased in a fluctuating variation with a notably increasing trend after 1994. With the changes in precipitation and temperature, the evapotranspiration, water requirement, irrigation water use and irrigation leaching amount showed a significant decreasing trend during 1961–2017 and 1961–1990, which experienced a substantial increase during 1991–2017. The water profit and loss index had a significant increasing trend during 1961–2017. Wind speed (WS) may have exerted the greatest influence on the variability in *D_cr_* and *IR* between 1961 and 1990, while sunshine hours (SH) may have played a more prominent role in explaining the variability in *D_cr_*. The precipitation (*Pre*) played a more important role in explaining the variability of *IR* from 1991 to 2017.

The climate of the Tarim basin was previously characterized by low precipitation and high evaporation. However, the climate has become a warmer and wetter climate in recent decades. Water resources are extremely indispensable and valuable in arid regions. Agricultural production highly depends on water availability. Practical and effective water resource management strategies must be carried out. This study provided important agricultural information for the adjustment of the cotton planting area, irrigation method and irrigation time with further global warming.

## Figures and Tables

**Table 1:** The partial correlation coefficients between *D_cr_*, *IR* and meteorological elements.

Variable	Time Period	Tmean	*Pre*	*RH*	WS	SH
*D_cr_*	1961–1990	0.17	−0.32	−0.36	0.92 **	0.68 **
	1991–2017	0.76 **	−0.41	−0.77 **	0.49 *	0.82 **
*IR*	1961–1990	0.20	−0.74 **	−0.40 *	0.83 **	0.65 **
	1991–2017	0.75 **	−0.92 **	−0.77 **	0.19	0.85 **

* *p* < 0.05; ** *p* < 0.01.

## Data Availability

All available data are reported in the paper.

## References

[1] Li X., Zhang S., Peng H., Hu X., Ma Y. (2013). Soil water and temperature dynamics in shrub-encroached grasslands and climatic implications: Results from Inner Mongolia steppe ecosystem of north China. Agric. For. Meteorol..

[2] Yu Y., Yu R., Chen X., Yu G., Gan M., Disse M. (2017). Agricultural water allocation strategies along the oasis of Tarim River in Northwest China. Agric. Water Manag..

[3] Geng Q., Zhao Y., Sun S., He X., Wang D., Wu D., Tian Z. (2023). Spatio-temporal changes and its driving forces of irrigation water requirements for cotton in Xinjiang, China. Agric. Water Manag..

[4] Zhang T., Zhai Y., Ma X., Shen X., Bai Y., Zhang R., Ji C., Hong J. (2021). Towards environmental sustainability: Life cycle assessment-based water footprint analysis on China’s cotton production. J. Clean. Prod..

[5] Jans Y., von Bloh W., Schaphoff S., Müller C. (2021). Global cotton production under climate change—Implications for yield and water consumption. Hydrol. Earth Syst. Sci..

[6] Liu Y., Qiao C. (2023). Study on evapotranspiration of cotton field under drip irrigation in oasis of arid region. Arid Zone Res..

[7] Lei C., Zhang-Lu Q., Xin-Lin H.E., Yan Y.U. (2018). The Effect of Drip Irrigation Under Mulch in the Process of Evapotranspiration in Oasis Cotton Fields. China Rural Water Hydropower.

[8] Wu C. (2016). Study on Evapotranspiration and Crop Coefficient of Cotton with Drip Irrigation under Mulch in Arid Oasis.

[9] Chen X., Qi Z., Gui D., Gu Z., Ma L., Zeng F., Li L. (2019). Simulating impacts of climate change on cotton yield and water requirement using RZWQM2. Agric. Water Manag..

[10] Yang Y., Yang Y., Han S., Macadam I., Liu D.L. (2014). Prediction of cotton yield and water demand under climate change and future adaptation measures. Agric. Water Manag..

[11] Su Y., Wang J., Li J., Wang L., Wang K., Li A., Gao L., Wang Z. (2023). Spatiotemporal changes and driving factors of reference evapotranspiration and crop evapotranspiration for cotton production in China from 1960 to 2019. Front. Environ. Sci..

[12] Benouniche M., Errahj M., Kuper M. (2016). The Seductive Power of an Innovation: Enrolling Non-conventional Actors in a Drip Irrigation Community in Morocco. J. Agric. Educ. Ext..

[13] He P., Zhang F., Fan J., Hou X., Liu X., Zhang Y., Xue Z. (2020). Effects of soil water Regulation on growth, quality and water use of cotton under drip irrigation in Southern Xinjiang. Agric. Res. Arid Areas.

[14] Zhao B., Wang Z., Li W. (2016). Effects of drip irrigation pattern and quota on soil water and salt distribution and cotton growth in winter irrigated cotton field in Northern Xinjiang. Trans. Chin. Soc. Agric. Eng..

[15] Yu Z., Wang L.H., Sun S.M., Chen X.L., Ji L.Y.L., Hu S.J. (2011). Indexes of salt tolerance of cotton in Akesu River Irrigation District. Sci. Agric. Sin..

[16] Zhang H., Yang P., Wang C., Li X. (2016). Effect of Winter Irrigation Amount on Soil Moisture and Salt Distribution in Arid Area. J. Irrig. Drain..

[17] Hu Q., Cao H., He Z., Ding B., Zhang Y. (2022). Combined Effect of Drip Irrigation Amount and Straw Mulch on Growth and Yield of Cotton in Salinized Soils in Northern Xinjiang. J. Irrig. Drain..

[18] Liu X., Yan F., Wu L., Zhang F., Yin F., Abdelghany A.E., Fan J., Xiao C., Li J., Li Z. (2023). Leaching amount and timing modified the ionic composition of saline-alkaline soil and increased seed cotton yield under mulched drip irrigation. Field Crops Res..

[19] Song X., Cao H., He Z., Ding B., Yao N. (2023). Applicability of the Aquacrop model in optimization of irrigation and salt leaching schedule during the reproductive period of cotton in Northern Xinjiang of China. Trans. Chin. Soc. Agric. Eng..

[20] Yan Y. (2016). Cotton water requirements and water saving benefit under mulched drip irrigation in Tarim Irrigated area. Res. Soil Water Conserv..

[21] Xu C., Lu C., Wang J. (2021). Impact of Meteorological factors on wheat growth period and irrigation water requirement—A case study of the Beijing-Tianjin-Hebei region in China. bioRxiv.

[22] Luo N., Bake B. (2017). Spatio-temporal variation of water requirement and meteorological impact factors of cotton in North Xinjiang, China. Chin. J. Appl. Ecol..

[23] Li Z., Chen Y., Yang J., Wang Y. (2014). Potential evapotranspiration and its attribution over the past 50 years in the arid region of Northwest China. Hydrol. Process..

[24] Chen Y., Deng H., Li B., Li Z., Xu C. (2014). Abrupt change of temperature and precipitation extremes in the arid region of Northwest China. Quat. Int..

[25] Dong Q., Wang W., Shao Q., Xing W., Ding Y., Fu J. (2019). The response of reference evapotranspiration to climate change in Xinjiang, China: Historical changes, driving forces, and future projections. Int. J. Climatol..

[26] Zhou H., Shen M., Chen J., Xia J., Hong S. (2018). Trends of natural runoffs in the Tarim River Basin during the last 60 years. Arid Land Geogr..

[27] Tao H., Gemmer M., Bai Y., Su B., Mao W. (2011). Trends of streamflow in the Tarim River Basin during the past 50 years: Human impact or climate change?. J. Hydrol..

[28] Gao X., Ye B., Zhang S., Qiao C., Zhang X. (2010). Glacier runoff variation and its influence on river runoff during 1961–2006 in the Tarim River Basin, China. Sci. China Earth Sci..

[29] Dinpashoh Y., Jhajharia D., Fakheri-Fard A., Singh V.P., Kahya E. (2011). Trends in reference crop evapotranspiration over Iran. J. Hydrol..

[30] Kousari M.R., Zarch M.A.A., Ahani H., Hakimelahi H. (2013). A survey of temporal and spatial reference crop evapotranspiration trends in Iran from 1960 to 2005. Clim. Chang..

[31] Zhao Q., Ye B., Ding Y., Zhang S., Yi S., Wang J., Shangguan D., Zhao C., Han H. (2012). Coupling a glacier melt model to the Variable Infiltration Capacity (VIC) model for hydrological modeling in north-western China. Environ. Earth Sci..

[32] Cao D., Yi X., Chen X., Xian J., Hu Q. (2022). Water Requirement of Non-Staple Crops in the Yellow River Delta Based on Climate Change. Chin. J. Soil Sci..

[33] Qi J., Batrrr B., Kalibir M. (2021). Climate Response of Water Demand Variation in Spring Wheat at Different Growth Stages in Northern Xinjiang. J. Triticeae Crops.

[34] Ren L., Ji J., Lu Z., Wang K. (2022). Spatiotemporal characteristics and abrupt changes of wind speeds in the Guangdong–Hong Kong–Macau Greater Bay Area. Energy Rep..

[35] Yang C.D., Xu M., Kang S.C., Fu C.S., Zhang W., Hu D.-D. (2024). Streamflow abrupt change and the driving factors in glacierized basins of Tarim Basin, Northwest China. Adv. Clim. Chang. Res..

[36] Diao W., Zhao Y., Dong Y., Zhai J., Wang Q., Gui Y. (2020). Spatiotemporal Variability of Surface Wind Speed during 1961–2017 in the Jing-Jin-Ji Region, China. J. Meteorol. Res..

[37] Kenett D.Y., Huang X., Vodenska I., Havlin S., Stanley H.E. (2015). Partial correlation analysis: Applications for financial markets. Quant. Financ..

[38] Poli D., Pastore V.P., Martinoia S., Massobrio P. (2014). Partial correlation analysis for functional connectivity studies in cortical networks. BMC Neurosci..

